
JOURNAL INFORMATION
==============================
NLM Title Abbreviation: Wirtschaftsdienst
Journal ID: Wirtschaftsdienst
NLM Journal ID: 101086612

Wirtschaftsdienst (Hamburg, Germany : 1949)

ISSN: 0043-6275

ARTICLE INFORMATION
==============================
PMCID: PMC7335916
PMID: 32834159
DOI: 10.1007/s10273-020-2681-8
Article ID: 2681
Article version: 1
Subjects: Ökonomische Trends

Wirkung der Corona-Krise auf die Staatsfinanzen

Impact of the Coronavirus Crisis on Public Finances

Gebhardt Heinz gebhardthg@gmx.de
1
Siemers Lars-H. Lars.Siemers@uni-siegen.de
2
1 Gneisenaustraße 93, 45472 Mülheim, Deutschland
2 grid.5836.8 0000 0001 2242 8751 Fakultät III Wirtschaftswissenschaften, Universität Siegen, Adolf-Reichwein-Straße 2a, 57076 Siegen, Deutschland
Electronic publication date: 2020 Jul 6
Print publication date: 2020
Volume: 100
Issue: 6
First page: 468
Last page: 470
Copyright: © Der/die Autor(en) 2020
License: Open Access Dieser Artikel wird unter der Creative Commons Namensnennung 4.0 International Lizenz veröffentlicht, welche die Nutzung, Vervielfältigung, Bearbeitung, Verbreitung und Wiedergabe in jeglichem Medium und Format erlaubt, sofern Sie den/die ursprünglichen Autor(en) und die Quelle ordnungsgemäß nennen, einen Link zur Creative Commons Lizenz beifügen und angeben, ob Änderungen vorgenommen wurden.
Die in diesem Artikel enthaltenen Bilder und sonstiges Drittmaterial unterliegen ebenfalls der genannten Creative Commons Lizenz, sofern sich aus der Abbildungslegende nichts anderes ergibt. Sofern das betreffende Material nicht unter der genannten Creative Commons Lizenz steht und die betreffende Handlung nicht nach gesetzlichen Vorschriften erlaubt ist, ist für die oben aufgeführten Weiterverwendungen des Materials die Einwilligung des jeweiligen Rechteinhabers einzuholen.
Weitere Details zur Lizenz entnehmen Sie bitte der Lizenzinformation auf http://creativecommons.org/licenses/by/4.0/deed.de.
License URL: https://creativecommons.org/licenses/by/4.0/

issue-copyright-statement: © The Author(s) 2020

==============================

Die Finanzlage des deutschen Staates verbesserte sich im vergangenen Jahrzehnt aufgrund günstiger gesamt- und finanzwirtschaftlicher Rahmenbedingungen stetig. Seit 2012 erzielte der Staat Budgetüberschüsse und die Staatsschuldenquote sank 2019 erstmals seit 2002 wieder unter die im Stabilitäts- und Wachstumspakt verankerte Schuldenobergrenze von 60 %. In diesem Jahr wird diese Entwicklung abrupt beendet – die Corona-Krise stürzt die deutsche Wirtschaft in die schwerste Rezession der Nachkriegszeit.

Aufgrund der komfortablen Finanzlage zu Beginn der Krise verfügt der Staat über ausreichende fiskalische Spielräume, um die automatischen Stabilisatoren wirken zu lassen und die negativen Folgen der Corona-Krise mit weitreichenden Rettungs- und Schutzschirmen abzufedern.[1] Dies wird die öffentlichen Haushalte aber außerordentlich belasten. Die Bundesregierung geht im Stabilitätsprogramm 2020 davon aus, dass die Defizitquote in diesem Jahr auf 7¼ % und die Schuldenquote auf 75¼ % steigen werden (BMF, 2020a). Diese Planungen beruhen auf dem Informationsstand bis Ende März 2020, der inzwischen überholt ist, da weitere fiskalpolitische Maßnahmen und ein umfassendes Konjunkturprogramm beschlossen wurden. Zudem umfasst das Stabilitätsprogramm noch keine Prognose der finanzwirtschaftlichen Entwicklung für 2021. Wir legen deshalb eine Prognose der staatlichen Finanzlage für 2020 und 2021 vor, die auf der Frühjahrsprojektion der Bundesregierung (Altmaier, 2020) basiert und die seit der Veröffentlichung des Stabilitätsprogramms beschlossenen Maßnahmen berücksichtigt.[2]

Um die rasante Ausbreitung des Coronavirus zu verlangsamen und eine Überlastung der Intensivstationen zu verhindern, sah sich die Bundesregierung im März 2020 gezwungen, umfängliche Beschränkungen des öffentlichen Lebens anzuordnen und die damit verbundenen volkswirtschaftlichen Kosten in Kauf zu nehmen. Auch viele andere Staaten haben umfassende Einschränkungen der wirtschaftlichen Aktivitäten beschlossen, um die Pandemie einzudämmen. Dies trifft die international stark verflochtene deutsche Volkswirtschaft in besonderem Maße. Die partielle Stilllegung der Wirtschaft, die Störung globaler Wertschöpfungsketten, Lieferengpässe bei Vor- und Zwischenprodukten, die geringere Verfügbarkeit von Arbeitskräften sowie die Verunsicherung von Investoren und Konsumenten dämpfen die gesamtwirtschaftliche Nachfrage und Produktion massiv. Die Bundesregierung prognostiziert in ihrer aktuellen Frühjahrsprojektion für 2020 einen Rückgang des preisbereinigten Bruttoinlandsprodukts (BIP) von 6,3 %. Sie geht davon aus, dass die zur Bekämpfung der Pandemie eingeführten Beschränkungen des öffentlichen Lebens schrittweise gelockert und aufgehoben werden und dass ein Wiederanstieg der Neuinfektionen verhindert werden kann. Lohnzuschüsse, Liquiditätshilfen für Unternehmen und das erweiterte Kurzarbeitergeld sollen eine Insolvenzwelle verhindern und den Anstieg der Arbeitslosigkeit begrenzen, sodass das für die konjunkturelle Erholung erforderliche Produktionspotenzial möglichst weit erhalten bleibt. Unter diesen Annahmen prognostiziert die Bundesregierung für 2021 einen Zuwachs des preisbereinigten BIP von 5,2 %.

Um die von der Corona-Krise verursachten negativen gesamtwirtschaftlichen Auswirkungen zu begrenzen, lässt die Bundesregierung zum einen die automatischen Stabilisatoren wirken und nimmt die hohen rezessionsbedingten Haushaltsbelastungen ohne Ausgleich hin. Zum

1 Die günstigen finanzwirtschaftlichen Ausgangsbedingungen zur Bewältigung der fiskalischen Folgen der Corona-Krise sind großteils Sonderfaktoren zu verdanken, die inzwischen weggefallen sind, künftig entfallen oder sich sogar umkehren (Gebhardt und Siemers, 2020).

2 Dieser Artikel wurde am 29. Mai 2020 eingereicht, als Umfang und Ausgestaltung des Konjunkturpakets noch nicht bekannt waren. Nach der Bekanntgabe des Konjunkturpakets haben wir seine finanzwirtschaftlichen Auswirkungen noch berücksichtigt; eine exakte Abschätzung war aufgrund der teils noch fehlenden Konkretisierungen indes nicht möglich. Generell gilt, dass Prognosen der Staatsfinanzen derzeit mit einem sehr hohen Maß an Unsicherheit verbunden sind, da sich die Rahmenbedingungen fortlaufend ändern; zudem sind kaum belastbare Daten zu den gesamt- und finanzwirtschaftlichen Folgen der Pandemie verfügbar.

Abbildung 1
**Bruttoinlandsprodukt (BIP), Produktionspotenzial (PP) und Produktionslücke (PL)**
jeweils preisbereinigt in Mrd. Euro bzw. in % des potenziellen BIP

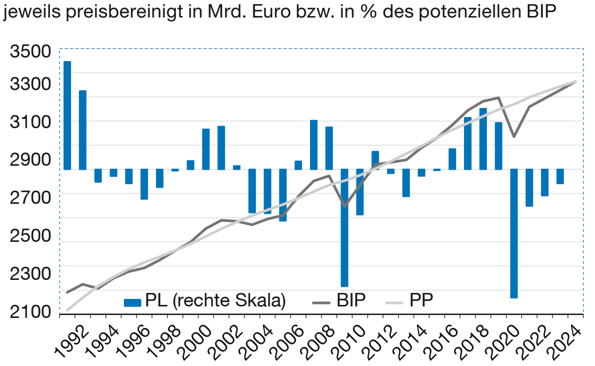

Ist: 1991 bis 2019; Prognose: 2020 bis 2024.

Quelle: Frühjahrsprojektion der Bundesregierung vom 29. April 2020 (BMWi, BMF 2020). Es ist zu beachten, dass in der Frühjahrsprojektion das erst am 3. Juni 2020 beschlossene Konjunkturpaket noch nicht berücksichtigt werden konnte. Durch dieses dürfte sich die Konjunktur günstiger entwickeln als damals prognostiziert. Die Deutsche Bundesbank (2020) beziffert in einer ersten groben Abschätzung auf Basis von Standardmodellelastizitäten die BIP-Effekte des Konjunkturpakets auf mehr als 1 % in diesem Jahr und ½ % im kommenden Jahr.

Abbildung 2
**Finanzierungssaldo, Konjunkturkomponente und struktureller Finanzierungssaldo des Staates**
in % des nomonalen BIP

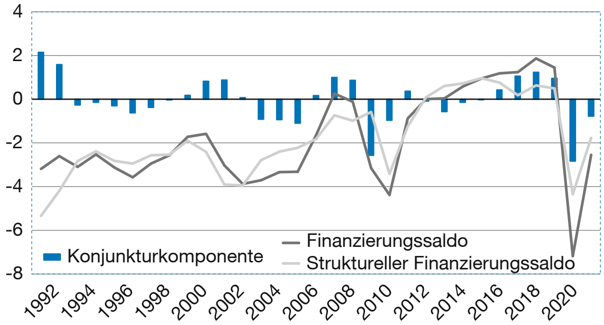

Ist: 1991 bis 2019; Prognose: 2020 bis 2021. Der Finanzierungssaldo 1995 berücksichtigt nicht die Vermögenstransfers im Zusammenhang mit der Übernahme der Schulden der Treuhandanstalt und der Wohnungswirtschaft der ehemaligen DDR (per saldo 119,6 Mrd. Euro).

Quelle: Angaben des Statistischen Bundesamts zur VGR, Frühjahrsprojektion der Bundesregierung und eigene Schätzung.

anderen stemmt sich die Finanzpolitik mit Rettungs- und Schutzschirmen von historischem Ausmaß und einem umfassenden Konjunkturprogramm gegen die Corona-Krise.

*Wirken der automatischen Stabilisatoren*: Die gesamtwirtschaftliche Produktionslücke beträgt 2020 nach den Schätzungen der Bundesregierung 5,3 % des potenziellen BIP (vgl. Abbildung 1). Die rezessionsbedingten Einbußen beim Steuer- und Beitragsaufkommen sowie die arbeitsmarktbedingten Mehrausgaben dürften sich 2020 auf insgesamt 93 Mrd. Euro belaufen. Die Konjunkturkomponente beträgt in Relation zum nominalen BIP 2,8 % und fällt damit um 0,3 Prozentpunkte höher aus als in der Finanz- und Wirtschaftskrise 2009 (vgl. Abbildung 2). Im kommenden Jahr wird das konjunkturbedingte Defizit voraussichtlich auf 27 Mrd. Euro bzw. 0,8 % des BIP sinken, da sich die hohe Unterauslastung der gesamtwirtschaftlichen Produktionskapazitäten aufgrund der konjunkturellen Erholung auf -1,5 % des Produktionspotenzials verringern dürfte.

*Diskretionäre Maßnahmen*: Um die medizinische Versorgung von Corona-Patienten sicherzustellen, Arbeitsplatzverluste und Unternehmensinsolvenzen möglichst eng zu begrenzen, und die Einkommen der privaten Haushalte zu stabilisieren, hat die Bundesregierung einen umfangreichen Rettungs- und Schutzschirm aufgespannt. Dieser umfasst eine Vielzahl von temporären einnahmen- und ausgabenseitigen Stützungsmaßnahmen (BMWi, 2020): unter anderem ein umfassendes Hilfsprogramm zur Sicherung der Liquidität von Unternehmen, Selbstständigen und Freiberuflern; Überbrückungshilfen (direkte Zuschüsse zu Betriebskosten) für „Solo-Selbstständige", Kleingewerbetreibende und Kleinunternehmer; die Stundung von Steuerzahlungen und die Anpassung der Vorauszahlungen bei den Ertragsteuern; den vorübergehend erleichterten Zugang zur Grundsicherung, zum Wohngeld und zum Kurzarbeitergeld; Ausgaben zum Ausbau der Intensivkapazitäten in den Krankenhäusern, zur Entwicklung von Präventionsmaßnahmen und zur Erforschung von Impfstoffen sowie weitere Vorhaben zur unmittelbaren Pandemiebekämpfung. Um etwaigen Mehrbedarf in den Einzelplänen aufgrund der Corona-Pandemie finanzieren zu können, wurde zudem eine globale Mehrausgabe in Höhe von 55 Mrd. Euro eingeplant. Die Haushaltsbelastungen werden im Nachtragshaushalt des Bundes für 2020 auf 122,5 Mrd. Euro veranschlagt.

Darüber hinaus sind seit der Verabschiedung des Nachtragshaushalts weitere Regelungen in Kraft getreten. Bei der Bundesagentur für Arbeit schlagen die bedingte Verlängerung der Bezugsdauer von Arbeitslosengeld I und die Erhöhung des Kurzarbeitergelds mit 2,1 Mrd. Euro (2020) und mit 0,6 Mrd. Euro (2021) zu Buche. Zudem haben auch die Länder zusätzliche Ausgaben beschlossen, die sich auf rund 60 Mrd. Euro belaufen. Zuletzt wurde für 2020 und 2021 ein Konjunkturpaket in Höhe von 130 Mrd. Euro beschlossen.[3]

3 Das Paket enthält eine Vielzahl an Maßnahmen. Die höchsten Belastungen entstehen durch Überbrückungshilfen für kleine und mittelständische Unternehmen (bis zu 25 Mrd. Euro), die temporäre Senkung der Mehrwertsteuersätze (20 Mrd. Euro), die vom Bund finanzierte Senkung der EEG-Umlage beim Strompreis (11 Mrd. Euro) sowie das Vorziehen von geplanten Aufträgen und Investitionen (bis zu 10 Mrd. Euro) (BMF 2020b). Die Maßnahmen machen 2020 einen zweiten Nachtragshaushalt erforderlich, der sich nach Bundesfinanzminister Scholz „aber in Grenzen halten (wird), da wir aus dem ersten Nachtragshaushalt noch 65 Mrd. Euro an Mitteln übrig haben" (Handelsblatt, 2020).

Ergänzend hat der Bund den Gewährleistungsrahmen im Bundeshaushalt um 356,5 Mrd. Euro auf 822 Mrd. Euro erhöht und den Wirtschaftsstabilisierungsfonds aufgelegt. Der Fonds hat ein Volumen von rund 600 Mrd. Euro und beinhaltet weitgehende staatliche Garantien, Rekapitalisierungsmaßnahmen und Kreditangebote, um Unternehmen bei der Sicherung ihrer Liquidität und der Abwehr einer Insolvenzgefahr zu unterstützen. Diese Maßnahmen haben auf den Finanzierungssaldo des Staates in der Abgrenzung des Vertrages von Maastricht nur begrenzt Einfluss, da sie nur dann defizitsteigernd zu Buche schlagen, wenn eine Zahlungspflicht des Staates entsteht.

Neben den automatischen Stabilisatoren tragen die diskretionären Maßnahmen dazu bei, die wirtschaftlichen Folgen der Corona-Krise abzufedern und die Konjunktur wieder in Schwung zu bringen. Die fiskalischen Impulse dieser Maßnahmen belaufen sich 2020 auf insgesamt 210 Mrd. Euro bzw. in Relation zum nominalen BIP auf 6,3 % und 2021 auf 40 Mrd. Euro (1,1 % des BIP). Dabei wird davon ausgegangen, dass die sehr umfangreiche globale Mehrausgabe des Nachtragshaushalts nur zum geringen Teil verausgabt wird und dass die Mittel aus dem Konjunkturpaket zu reichlich zwei Dritteln 2020 und zu knapp einem Drittel 2021 abfließen. Hierdurch werden die öffentlichen Haushalte indes nicht in gleichem Umfang belastet; die Rettungs- und Schutzschirme und das Konjunkturpaket führen zu Wachstums- und Beschäftigungseffekten, aus denen höhere Steuer- und Beitragseinnahmen sowie geringere arbeitsmarktbedingte Ausgaben resultieren. Alles in allem dürfte sich das staatliche Budgetdefizit 2020 auf 235 Mrd. Euro bzw. 7,2 % des nominalen BIP belaufen (vgl. Abbildung 2). 2021 wird es aufgrund der konjunkturellen Erholung, des Auslaufens der Maßnahmen aus den Rettungs- und Schutzschirmen und der geringeren Impulse aus dem Konjunkturpaket deutlich sinken – voraussichtlich auf 89 Mrd. Euro (2,5 % des BIP).

Auch die um konjunkturelle Einflüsse bereinigte, strukturelle Finanzlage des Staates wird sich erheblich verschlechtern.[4] Nach acht Jahren mit strukturellen Überschüssen wird der Staat 2020 wegen der Corona-Krise ein strukturelles Defizit von 142 Mrd. Euro (4,3 % des nominalen BIP) hinnehmen müssen (vgl. Abbildung 2). 2021 dürfte das strukturelle Defizit auf 62 Mrd. Euro (1,8 % des nominalen BIP) sinken, da die diskretionären Maßnahmen aus den Rettungs- und Schutzschirmen auslaufen und die Impulse aus dem Konjunkturpaket merklich geringer ausfallen als 2020.

Als Folge der hohen Neuverschuldung der Gebietskörperschaften,[5] der zusätzlichen Kredite und Beteiligungen von rund 280 Mrd. Euro und des Rückgangs des nominalen BIP wird die Staatsschuldenquote 2020 auf etwa 77½ % hochschnellen. Sollte es zu einer zweiten Ansteckungswelle mit einem erneuten Shutdown kommen, muss mit einem noch kräftigeren Anstieg der Schuldenquote gerechnet werden. Neben weiteren erheblichen konjunkturbedingten Haushaltsbelastungen käme es zu zusätzlichen Unternehmensinsolvenzen, welche die öffentlichen Haushalte aufgrund der Kreditausfälle und der übernommenen Bürgschaften belasten würden.

Um die langfristige Tragfähigkeit der Staatsschulden zu sichern, steht nach der konjunkturellen Erholung ein schwieriger Konsolidierungsprozess an, zumal der Staat – anders als im vergangenen Jahrzehnt – nicht mehr von günstigen Sonderfaktoren profitieren wird. Der demografische Wandel wird künftig vielmehr zu steigenden altersabhängigen Ausgaben führen, und bei Normalisierung der Geldpolitik werden die Aufwendungen für den Schuldendienst steigen (Gebhardt und Siemers, 2020). Daher wird die Finanzpolitik nach der Krise gefordert sein, die Ausgaben konsequent an den verfügbaren strukturellen Einnahmen zu orientieren.

4 Der strukturelle Finanzierungssaldo wird durch Subtraktion der geschätzten Konjunkturkomponente von dem um Einmaleffekte bereinigten Finanzierungssaldo berechnet. Die Konjunkturkomponente ergibt sich aus dem Produkt der Produktionslücke und der von der Europäischen Kommission (2019) berechneten Budgetsemielastizität. Da diese in der Corona-Krise höher ausfallen dürfte als von der EU-Kommission berechnet, unterliegen unsere Berechnungen der Konjunktur- und Strukturkomponente einer gewissen Unschärfe.

5 Die Defizite der Sozialversicherungen schlagen beim Schuldenstand nicht zu Buche, da sie durch Rücklagen ausgeglichen werden.

Heinz Gebhardt, Dipl.-Volkswirt, ist wirtschaftspolitischer Berater und war wissenschaftlicher Mitarbeiter am RWI Essen sowie Mitglied im Arbeitskreis Steuerschätzungen.

PD Dr. Lars-H. Siemers ist wirtschaftspolitischer Berater und Hochschuldozent am Lehrstuhl für Europäische Wirtschaftspolitik an der Universität Siegen.


Literatur

Altmaier, P. (2020), Schwere Rezession durch die Corona-Pandemie, Schlaglichter der Wirtschaftspolitik, Ausgabe Mai, 10–17.
Bundesministerium der Finanzen (BMF) (2020a), Deutsches Stabilitätsprogramm 2020, Berlin, 22. April, 18.
Bundesministerium der Finanzen (BMF) (2020b): Corona-Folgen bekämpfen, Wohlstand sichern, Zukunftsfähigkeit stärken, Ergebnis Koalitionsausschuss 3. Juni.
Bundesministerium für Wirtschaft und Energie (BMWi) (2020), Schutzschirm aufgespannt, Schlaglichter der Wirtschaftspolitik, Ausgabe Mai, 20–29.
Bundesministerium für Wirtschaft und Energie (BMWi); Bundesministerium der Finanzen (BMF) (2020), Gesamtwirtschaftliches Produktionspotenzial und Konjunkturkomponenten, Frühjahrsprojektion der Bundesregierung vom 29. April.
Deutsche Bundesbank (2020), Zum angekündigten Fiskalpaket der Koalitionsparteien, Monatsbericht, Juni, 17.
Europäische Kommission (2019), Report on Public Finances in EMU 2018: Recent developments in the fiscal surveillance framework, Institutional Paper, 095, Januar.
Gebhardt, H. und L.-H. Siemers (2020), Die Staatsfinanzen in der Corona-Krise: günstige Bedingungen garantieren die staatliche Handlungsfähigkeit, Wirtschaftsdienst, 100(7), im Erscheinen.10.1007/s10273-020-2695-2 PMC7368592 32834167
Handelsblatt (2020), Alle werden zufrieden sein, 5. Juni 2020.
